# Research on Real-Time Robust Optimization of Perishable Supply-Chain Systems Based on Digital Twins

**DOI:** 10.3390/s23041850

**Published:** 2023-02-07

**Authors:** Yingnian Wu, Jing Zhang, Qingkui Li, Hao Tan

**Affiliations:** 1 School of Automation, Beijing Information Science and Technology University (BISTU), Beijing 100192, China; 2 Institute of Intelligent Networked Things and Cooperative Control, Beijing Information Science and Technology University (BISTU), Beijing 100192, China; 3 Intelligent Perception and Control of High-End Equipment Beijing International Science and Technology Cooperation Base, Beijing Information Science and Technology University (BISTU), Beijing 100192, China

**Keywords:** digital twins, perishable supply chain, complex environments, real-time robust optimization, *H*_∞_ controller, uncertain system

## Abstract

Aiming at the real-time robust optimization problem of perishable supply-chain systems in complex environments, a real-time robust optimization scheme based on supply-chain digital twins is proposed. Firstly, based on the quantitative logical relationship between production and sales of single-chain series supply-chain system products, the state space equation of the supply-chain system with logical characteristics, structural characteristics, and quantitative characteristics was constructed, and twin data were introduced to construct the digital twins of supply chains based on the state-space equation. Secondly, the perishable supply-chain system in complex environments was regarded as an uncertain closed-loop system from the perspective of the state space equation, and then a robust $H_{\infty }$ controller design strategy was proposed, and the supply-chain digital twins was used to update and correct the relevant parameters of the supply-chain system in real-time, to implement the real-time robust optimization based on the supply-chain digital twins. Finally, the simulation experiment was carried out with a cake supply-chain production as an example. The experimental results show that the real-time updating of relevant parameters through the digital twins can help enterprise managers to formulate reasonable management plans, effectively avoid the shortage problem of enterprises in the cake supply-chain system, and reduce the maximum inventory movement standard deviation of each link by 12.65%, 6.50%, and 14.87%, and the maximum production movement standard deviation by 70.21%, 56.84%, and 45.19%.

## 1. Introduction

The supply-chain system is a cascade system composed of multiple links, and its upstream and downstream enterprises are committed to selling products to consumers at the lowest operating costs and the fastest speed. With the trend of globalization, more and more enterprises and researchers pay attention to the management and optimization of the supply-chain system, especially from the perspective of production–storage–sales [1,2,3,4,5], the supply-chain system to optimize management. In the modern supply-chain system, the operating environment of the supply-chain system is complex, and the challenges it faces include many aspects such as natural disasters [6,7], public health [8,9,10], and wars [11,12,13].These will lead to changes in the market environments, causing market demand fluctuations.Especially in recent years, the emergence of crises such as coronavirus disease 2019 (COVID-19)and the Russian–Ukrainian war put the world supply-chain system in a state of extreme complexity, high uncertainty, and disruption. Companies may suffer significant financial losses if they are unable to quickly and effectively respond to changes in the market environments and adapt to the complex supply chain system environments.For example, the COVID-19 outbreak at the start of 2020 made many fresh products unmarketable, resulting in a wide spectrum of product damage and expiry and a significant waste of resources and economic damages. This shows that especially for the supply-chain system of perishable products, if the product is unsellable or transportation is interrupted due to changes in the complex market environments, it will cause product waste and cause huge economic losses. This also makes the managers aware that traditional supply-chain management techniques are no longer adequate to manage the complex market environments. Instead, effective management techniques are urgently required to assist enterprises in managing the complex market environments and developing a reasonable production–storage–sales strategy to increase the robustness and anti-disturbance capability of the supply-chain system.

The control of the supply-chain system is a management method that combines the logical relationship of the number of purchases, sales, and inventory of each link in the complete supply-chain system, combines the structural characteristics, constructs the dynamic model of the supply-chain system in the case of considering the cycle, and reasonably designs the control method based on the dynamic model, to achieve the ideal state of production, demand, shipment, inventory, and other parameters. At the same time, due to the suddenness and unpredictability of the sudden state, it is also necessary for the real-time optimization management of the supply-chain system. According to the real-time operation of the supply-chain system, a reasonable production-storage-sales scheme is formulated, and the digital twin technology can well meet the demand of real-time optimization. The digital twin technology is used to build digital twins of the supply chain, and then the digital twins is used to realize the real-time connection between the physical entities of the supply chain and the virtual simulation environment, so as to realize the real-time robust optimization of the supply-chain system. The existing research closely related to this paper mainly focuses on three aspects: the application research of automation control theory in supply-chain system, the application research of robust $H_{\infty }$ state feedback control, and the application of digital twin technology in supply-chain management.

With the development of the automotive control theory, more and more scholars are also focusing on the cross-field application of automation technology, including the application in the field of supply-chain management. At present, the research on the application of automation technology in the field of supply chain management mainly focuses on two aspects.

- Application of frontier automated intelligent algorithms to manage and optimize the supply-chain system [14,15,16,17]. The research in this aspect is to create an objective optimization function based on the supply-chain system, solve it using an intelligent algorithm, and provide a management and optimization strategy for the supply-chain system. The advantage of this research is that it can help the supply-chain system to achieve multi-objective optimization, but the disadvantage is that the optimal solution is often a range solution, which cannot give business managers clear and accurate production, purchase, and shipment strategies. At the same time, it requires relatively high model accuracy, which makes it necessary to set a lot of assumptions in the model construction process.
- Application of automation control theory for supply-chain system management and optimization [18,19,20,21]. This research is based on the existing supply-chain production and marketing model, taking inventory and operation costs as the controlled objects and constructing a supply-chain system model with reasonable assumptions, and then applying control theory ideas to optimize the management of the supply-chain system based on the model. Compared with the intelligent algorithmic solutions, the control theoretic can help managers to develop clear and accurate production–storage–shipment strategies that do not require high model accuracy.

Due to the unique advantages of control philosophy in supply-chain management, more and more scholars and business managers have conducted related research. Simon [22], a an American scholar, was the first to apply the idea of control systems to inventory management. He viewed the inventory optimization problem as a control system and applied the Laplace transform to convert the differential equation into the transfer function of the control system. Vladimir et al. [23] considered the optimization problem of a failure-prone manufacturing system with uncertainty in demand and inventory levels, and designed a controller based on adaptive control for the online estimation and optimal control of a supply-chain inventory system in the presence of unknown demand and inaccurate inventory. Bartoszewicz et al. [24] used sliding mode control to optimize the management of manufacturers’ inventories with storage constraints for manufacturers’ inventory management under different demands. Achamrah et al. [25] used the genetic algorithm and deep reinforcement learning to solve the inventory path problem with transit and substitution under dynamic and stochastic demand. Such literature addresses the problem of demand uncertainty in complex environments from a control perspective, but ignores the fact that complex environments can cause not only changes in demand but also changes in the structural parameters of the supply-chain system, which is not considered in this literature. This is one of the motivations for this paper. Of course, some scholars have also focused on the study of supply-chain inventory management with parameter uncertainty. Chen et al. [26] studied the optimization of supply-chain product renewal based on a multi-period model and fuzzy control in an uncertain environment, addressing the problem of how to satisfy uncertain market demand in the case of manufacturer product improvement. David et al. [27] studied the design of a multi-configuration logistics network with capacity constraints under perturbations and parameter uncertainties using a pharmaceutical supply chain as an example of a retailer in direct contact with consumers. However, such literature does not take into account the problem of product self-loss and production conformity in the case of multiple cycles and does not study perishable supply-chain systems that are subject to environmental impacts, which is another motivation for this paper.

$H_{\infty }$ robust control has a better control impact for uncertain systems in complicated contexts when compared to other automated control approaches. The main purpose of $H_{\infty }$ robust control is to address the issue of uncertainty disturbance of the controlled object. By designing $H_{\infty }$ robust control, it is possible to improve the stability and quality robustness of the system, which makes $H_{\infty }$ robust control a widely used model for solving the problems of an unknown control system. Indri et al. [28] applied of robust $H_{\infty }$ control to the industrial robotic arm to solve the tracking problem of the robotic arm under the action of unknown disturbances. Xu et al. [29] studied the quadrotor UAV flight problem inside a culvert, and used robust $H_{\infty }$ control based on the state observer for dynamic planning of UAV flight state inside a culvert. Wang et al. [30] used the $H_{\infty }$ robust control strategy to solve the wind turbine hydraulic pitch system control problem, which greatly reduced the system error of wind turbines. In addition to the above industrial applications, $H_{\infty }$ robust control has also been applied in the field of supply-chain inventory management, but the current research in the field of supply-chain inventory management mainly focuses on solving the bullwhip effects problem [31,32,33], and there is no research related to the application of perishable supply-chain systems in complex environments. This is the third research motivation for this paper. Although $H_{\infty }$ robust control can successfully solve the uncertain system control problem under unknown environments, the fluctuation range of unknown parameters is frequently determined by using data statistics or expert experience, which cannot be dynamically changed in real-time with time and environmental changes, while the digital twin model can obtain the relevant parameter data in real-time based on physical entities, and then dynamically update the simulation.

The concept of the digital twin was first proposed by professor Grieves [34] in 2003 at the university of Michigan in the United States, and it was first used in the aerospace and defense sectors, considerably enhancing their growth and production. Since then, several industries have adopted and developed digital twin technology. Initially, the digital twin model followed the three-dimensional model proposed by professor Grieves [34] for model building, until 2019, when Tao Fei et al. [35] proposed a five-dimensional digital twin model, which introduced data and services into the model and greatly enriched the model structure and extended the application area of digital twin technology. Through the continuous discussion of the concept and definition of the digital twin, it is clear that the objects it applies to different fields are different, among which digital twins refers to whole physical entities and their digital drives [36]. At present, the applications of digital twin technology in supply-chain systems are mostly focused on product design [37], shop floor monitoring and forecasting [38], and product line management [39]. However, the applicability to multi-cascade supply-chain systems has not been thoroughly studied, which is the final research objective for this article. Considering the current technology, there are two main problems: the construction of virtual entities is relatively complex [40] and data collection between multiple enterprises is relatively difficult [41]. However, if we build the digital twin using a dynamic equation, it can accurately reflect the logical, structural, and quantitative characteristics of the supply-chain system, avoiding the mentioned problems. It can also be used to obtain real-time access to the operational state of supply-chain system and to carry out robust real-time supply-chain optimization.

Based on the above analysis, this paper designs the $H_{\infty }$ robust controller for perishable supply-chain systems in complex environments and builds the digital supply-chain twins to obtain unknown parameters in real-time to achieve the dynamic robust optimization of the supply-chain system. The goal of this work is to resolve the control problem of perishable supply-chain inventory systems under the fluctuation of market demand caused by complex environments, and supply-chain uncertainty. It can also help the supply-chain system to adapt to the complex market environments and meet unknown demands, while reducing the impact of demand fluctuation and parameter changes on the operating cost of the supply-chain system. The rest of this paper is organized as follows. Section 2 builds supply-chain digital twins based on the supply-chain state-space equation by digital twin technology. We design a robust $H_{\infty }$ controller of the supply-chain system in complex environments and illustrate the real-time robust optimization principle based on the supply-chain digital twins in Section 3. Section 4 discusses simulation and comparison experiments based on the cake supply-chain system. We make conclusions and discuss future possibilities in Section 5.

## 2. Supply-Chain Digital Twins

The supply-chain system is a complex network system made up of multi-level links, and because of its structural complexity, high level of unpredictability, and high operating expenses, researchers and business managers have faced several challenges while designing and validating algorithms. Firstly, if algorithm verification is carried out in the real supply-chain system, a lot of resources will be wasted. Secondly, because the operating parameters of supply-chain system are frequently influenced by environmental factors, setting them beforehand based on simulation results will result in an inaccurate description of the supply-chain system and inaccurate experimental results. Finally, owing to the unexpectedness and unpredictability of emergencies, there will be some uncertainty in the algorithm’s verification if the pertinent data from the supply-chain system cannot be obtained in real-time.

**Figure 1:**
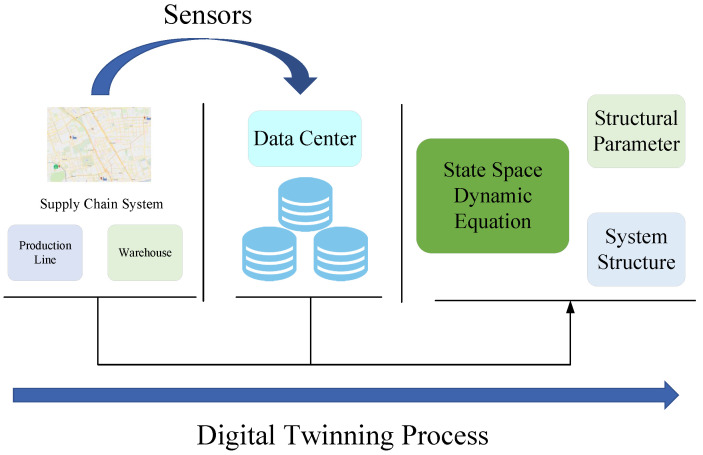
Digital twinning process of supply-chain system.

To solve the previous issues, this paper builds digital twins for real-time data transmission to better monitor the operating status of the supply-chain system in complex environments, and then uses real-time data to correct unknown parameters in real-time, allowing for the development of more reasonable production plans can be made that are more reasonable for a variety of operating conditions and environments for perishable supply-chain systems in complex environments. This lowers inventory costs and increases the enterprise’s robustness and anti-disruption capabilities, while it can also meet market demand. The supply-chain digital twinning process is shown in Figure 1.

From Figure 1, we can see that the process of supply-chain digital twinning in this paper is divided into two steps: creating the state-space equation of supply-chain system, which includes structural and parametric properties, and creating its digital twins using the state-space equation.

### 2.1. Dynamics Equation of Perishable Supply-Chain System

**Figure 2:**
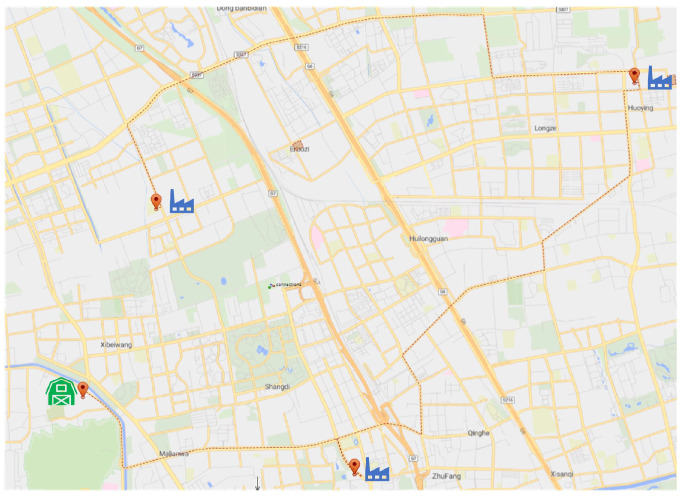
Single-chain series supply-chain system.

**Figure 3:**
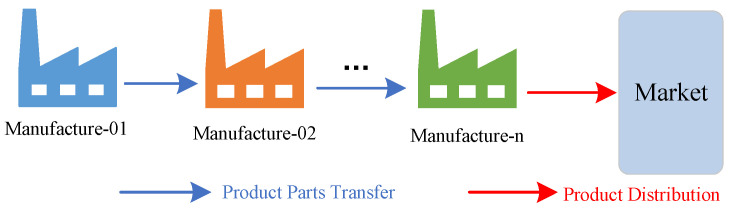
Equivalent diagram of single-chain tandem supply-chain system.

We base our analysis on a single-chain tandem supply-chain system, the structure of which is shown in Figure 2. In a single-chain tandem supply-chain system, only the last producer needs to sell directly to the market. At the same time, in most cases, all parts of the supply-chain system are replenished according to a certain production cycle. According to the definition of single-chain tandem supply-chain system and the schematic diagram of Figure 2, it can be equated as shown in Figure 3.

As shown in Figure 3, manufacturer-01 creates components for transfer to manufacture-02, manufacture-02 creates components for transfer to downstream producers, and producer-n creates finished items for sale. In order to model and analyze the single-chain tandem supply-chain system more accurately, so as to be closer to the operation state of each link of the supply-chain system in the real environment, the following assumptions need to be made.

**Assumption** **1.** *This paper considers the situation that the supply-chain system is a single chain. Only the last-link manufacturer directly outputs products to the market. Other links do not export goods to the outside market, only produce product parts for transmission, and only transmit within the supply-chain system.*

**Assumption** **2.** *In this paper, we consider the production replenishment time point of the supply-chain inventory system is kT, where k=0,1,2,…,n, T represents the production planning cycle. In most cases, the production planning cycle is day, so this paper uses k to represent kT.*

**Assumption** **3.** *The manufacturer’s production is influenced by various factors such as production capacity and production equipment, and it cannot guarantee that all the parts produced are qualified. In this paper, we set the production rate in a single cycle T as δ, and then we obtain*

(1) $$\begin{array}{c} \begin{array}{c} \delta ={\left[\delta_{1},\delta_{2},\ldots ,\delta_{n}\right]}^{T}. \end{array} \end{array}$$

**Assumption** **4.** *The product under consideration is perishable, and there is a self-loss of inventory at each stage of the production process due to the product itself or storage conditions, and assume that the self-loss rate of the supply-chain inventory system is ρ for a single cycle T, then we can obtain*

(2) $$\begin{array}{c} \begin{array}{c} \rho ={\left[\rho_{1},\rho_{2},\ldots ,\rho_{n}\right]}^{T}. \end{array} \end{array}$$

**Figure 4:**
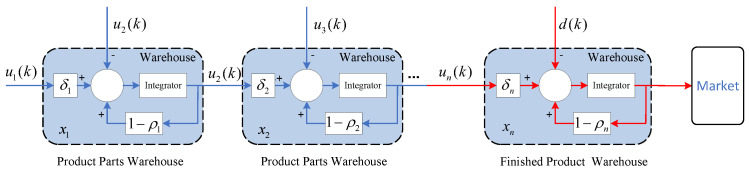
Equivalent diagram of single-chain tandem supply-chain system.

According to the internal linkage of each link in the sales route of the supply chain system and the logical relationship between production and sales of the products sold as shown in Figure 3, the most important inventory quantity of each link of the supply-chain system is taken as the controlled object, and the inventory relationship of each link of the supply-chain system can be obtained by equivalence and simplification based on the above assumptions, as shown in Figure 4.

Where $u_{i}$ denotes the planned production of the *i*th manufacturer; $x_{i}$ represents the parts inventory of the *i*th manufacturer; $\delta_{i}$ indicates the production pass rate parameter of the *i*th manufacturer in the supply chain system; $\rho_{i}$ means the inventory self-loss rate of the *i*th manufacturer in the supply-chain system; *d* denotes the fixed market demand.

Firstly, based on the above assumptions and considering the logical relationship between the parameters of the single-chain tandem supply-chain system, the inventory relationship between the producers in each link of the supply-chain system in Figure 4 can be expressed as follows.

(3) $$\begin{array}{c} x_{1}\left(k+1\right)=\left(1-\rho_{1}\right)x_{1}\left(k\right)+\delta_{1}u_{1}\left(k\right)-u_{2}\left(k\right)\\ x_{2}\left(k+1\right)=\left(1-\rho_{2}\right)x_{2}\left(k\right)+\delta_{2}u_{2}\left(k\right)-u_{3}\left(k\right)\\ \;\;\;\;\;\;\;\;\;\;\;\;\;\;\;\;\;\;\;\vdots \\ x_{n}\left(k+1\right)=\left(1-\rho_{n}\right)x_{n}\left(k\right)+\delta_{n}u_{n}\left(k\right)-d\left(k\right) \end{array}$$

Secondly, based on Figure 4, the relationship between the inventory of each link of the supply-chain system, considering the production quantity, inventory quantity, and market demand, which are the most important concerns of enterprise managers, we let

(4) $$\begin{array}{c} \begin{array}{cc} & x\left(k\right)={\left[x_{1}\left(k\right),x_{2}\left(k\right),\ldots ,x_{n}\left(k\right)\right]}^{T}\in R^{n}\\ & u\left(k\right)={\left[u_{1}\left(k\right),u_{2}\left(k\right),\ldots ,x_{n}\left(k\right)\right]}^{T}\in R^{n}\\ & d\left(k\right)={\left[0,0,\ldots ,d\right]}^{T}\in R^{n}, \end{array} \end{array}$$

where x(k) denotes the set of inventory of each producer in the supply chain system at time *k*. u(k) represents the production quantity of each producer in the supply-chain system at time *k*. d(k) indicates the fixed market demand in the supply-chain system at time *k*.

Finally, by combining Equation (3) with Equation (4), the following Equation (5) can be obtained.

(5) $$\begin{array}{c} x_{i}\left(k+1\right)=Ax_{i}\left(k\right)+Bu_{i}\left(k\right)+Dd\left(k\right) \end{array}$$

where

$$A=\left[\begin{array}{cccccc} 1-\rho_{1} & 0 & 0 & \cdots & 0 & 0\\ 0 & 1-\rho_{2} & 0 & \cdots & 0 & 0\\ \vdots & \vdots & \vdots & \ddots & \vdots & \vdots \\ 0 & 0 & 0 & \cdots & 1-\rho_{n-1} & 0\\ 0 & 0 & 0 & \cdots & 0 & 1-\rho_{n} \end{array}\right]$$

$$B=\left[\begin{array}{cccccc} \delta_{1}\; & \;\;-1 & 0 & \ldots & 0 & 0\\ 0 & \;\;\delta_{2}\; & \;\;-1 & \ldots & 0 & 0\\ \vdots & \vdots & \vdots & \ddots & \vdots & \vdots \\ 0 & 0 & 0 & \ldots & \delta_{n-1}\; & \;\;-1\\ 0 & 0 & 0 & \ldots & 0 & \;\;\delta_{n} \end{array}\right]$$

$$D={\left[\begin{array}{c} \;0\\ \;\vdots \\ \;0\\ -1 \end{array}\right]}_{n}.$$

In summary, the state-space equation for a single-chain tandem perishable supply-chain system with *n* producer links can be built as

(6) $$\begin{array}{c} \left\{\begin{array}{c} x(k+1)=Ax(k)+Bu(k)+Dd(k)\\ y(k)=Cx(k) \end{array}\right. \end{array}$$

where $y\left(k\right)\in R^{n}$ denotes the measurable output of the supply-chain system, $A\in R^{n\times n}$, $B\in R^{n\times n}$, $C\in R^{n\times n}$ are known constant matrices, respectively, and C is the unit matrix. We suppose that the (A,B) matrix is controllable and the (A,C) matrix is observable.

For the constructed dynamic equation of the single-chain tandem perishable supply chain system, the following remarks are needed.

**Remark** **1.** *The value range of the inventory level of each link in the supply-chain system is $x_{j}\left(k\right)⩽m_{j}$, where $m_{j}$ represents the maximum storage capacity of the warehouse in the j link. Of course, when the inventory is $x_{j}\left(k\right)<0$, it means that the link is currently in a short shortage state, which is also existing and reasonable for the real supply-chain system.*

**Remark** **2.** *The production of manufacturer has a certain range of values, which is $0<u_{1}\left(k\right)⩽{\bar{u}}_{max}$ and the ${\bar{u}}_{max}$ represents the maximum production capacity of the manufacturer.*

**Remark** **3.** *The fixed market demand faced by the supply-chain system is known to the upstream and downstream enterprises and is relatively stable, and the changes in market demand affecting the supply-chain are mainly caused by demand fluctuations.*

We set the market demand fluctuation ω(k) at time *k* based on the dynamic equation of the single-chain tandem perishable supply-chain system. We know that although the market demand fluctuation directly acts on the last producer, due to the bullwhip effects and the cascade effect of the single-chain supply-chain system, the market demand fluctuation will gradually affect the upstream producers in the supply chain against the direction of product flow. We let the impact matrix of market demand fluctuation ω(k) on the supply-chain system be $D_{1}\in R^{n}$, then the state-space equation of the supply-chain system with demand fluctuation can be obtained as shown in Equation (7).

(7) $$\left\{\begin{array}{c} x\left(k+1\right)=Ax\left(k\right)+Bu\left(k\right)+Dd\left(k\right)+D_{1}\omega \left(k\right)\\ y(k)=x(k) \end{array}\right.$$

To reflect the change of inventory costs in the supply-chain system, here we introduce the inventory costs parameter z(k) at the time *k*. The inventory cost z(k) of the supply-chain inventory system is closely related to the inventory quantity in each link of the supply chain. In this paper, let the matrix of inventory cost and inventory quantity related parameters be $C_{1}\in R^{1\times n}$; then, the state-space expression of the single-chain tandem perishable supply-chain system with inventory cost can be obtained as follows.

(8) $$\begin{array}{c} \left\{\begin{array}{c} x\left(k+1\right)=Ax\left(k\right)+Bu\left(k\right)+Dd\left(k\right)+D_{1}\omega \left(k\right)\\ y(k)=x(k)\\ z\left(k\right)=C_{1}x\left(k\right) \end{array}\right. \end{array}$$

### 2.2. Supply-Chain Digital Twins Based on State-Space Equation

**Figure 5:**
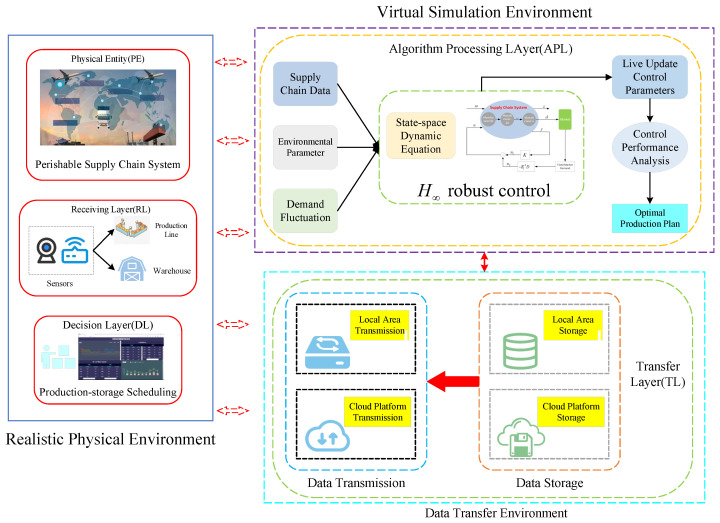
Digital twins of perishable supply-chain.

The digital twins of the supply chain established in this paper need to achieve the information transfer between the physical and virtual entities of the supply chain and their real-time interaction, and play a role in the whole process of robust optimization, so the digital twins built in this paper are shown in Figure 5, which mainly consists of the real physical environment, the virtual simulation environment, and the data transfer environment. The real physical environment consists of the physical entity layer, the data acquisition layer, and the production decision layer. The virtual simulation environment and the data transfer environment include the algorithm processing layer and the data transfer layer.

The real-world physical environment, the computer-generated simulation environment, and the data-dump layer are defined as follows from Figure 5.

- The physical entity layer is a perishable supply-chain system, which consists of several segments that produce perishable products, each of which includes production, storage, and sales components. During the operation of the supply-chain system, different types of data are generated, including supply-chain parameters, production data, and product storage data, which are transmitted to the data acquisition layer.
- The data acquisition layer is the medium through which the digital twins collects data, mainly the operational data of the supply-chain system, including production data and store data, and the external data of the supply-chain system, including weather, average temperature, and market demand.
- The production decision layer will create the best production plan based on market demand and the production plan generated by the algorithm processing layer, and then issue the production order to the terminal equipment to guarantee the supply-chain system runs normally.
- The algorithm processing layer, which is the central component of the supply-chain digital twins, directly sets the production strategy for each link in the system. To assist businesses in creating the best production plan, it first builds the supply-chain state-space equation based on data from the data receiving layer and then updates the operation state and pertinent supply-chain system parameters with the real-time transmitted data.
- The data transfer layer is mainly composed of data storage and data transmission, which realizes the efficient transfer of data between each link of the supply-chain system, and stores and recalls these data in real-time through a combination of cloud and local terminals.

By the supply-chain digital twins, we can realize the supply-chain system to link with the virtual simulation environment through the twin data in real-time to achieve better control effects, optimize the simulation results in the virtual environment, and help the enterprises in each link of the supply-chain system to make better production plans.

## 3. Analysis and Methods

The different operating environments of the supply-chain system are reflected in the state-space equation of the supply-chain system, which can be represented by different system parameters.The different operating environments of the supply-chain system are reflected in the state-space equation of the supply-chain system, which can be represented by different system parameters, and then the uncertain supply-chain system state-space equation can be used to describe the supply-chain system in a complex environments.

After describing the supply-chain system in complex environments by using the uncertain state-space equation, we propose the design theorem of a robust $H_{\infty }$ controller for an uncertain system, and then design the robust $H_{\infty }$ controller to meet the performance requirements, and finally use the supply-chain digital twins to obtain the unknown parameters of the supply chain system in the current environment in real-time to achieve the real-time robust optimization of the supply-chain system.

### 3.1. Model Analysis of Perishable Supply-Chain System in Complex Environments

The challenges that the modern supply-chain system faces emerge from a variety of sources, and the environment in which it operates is very complex. In particular, for the perishable supply-chain system, the unique characteristics of the items would create huge economic loss if the supply chain system was unable to address the issues and adapt to the complicated operational environments.Therefore, there is a pressing need to address the issue of how to improve the robustness of supply-chain system while coping with the complicated and ever-changing operational environments. Due to the complexity of the market and operating environments, the operation state of the supply-chain system will also change, which is reflected in the constructed state-space equation of the supply-chain system and can be expressed as the change of relevant parameters.We need to make some remarks about the perishable supply-chain system’s parameter changes in this paper because of the complex environments.

**Remark** **4.** *The dynamic model of the perishable supply-chain system is mainly characterized by two changes in parameters: internally, the self-loss rate ρ and the production conformity rate δ. Externally, it is mainly reflected in the changes in market demand fluctuation ω.*

The following assumptions on the influences on the supply-chain system need to be established in order to study the supply-chain system in the complex environments.

**Assumption** **5.** *The matrix $D_{1}$ of the impact of demand volatility on the supply-chain system and the parameter $C_{1}$ of the correlation between the supply-chain inventory quantity and inventory costs do not change under complex environments.*

**Assumption** **6.** *In complex environments, the fixed market demand of the supply-chain system does not change, but the change in market demand is mainly caused by the fluctuation of demand.*

**Figure 6:**
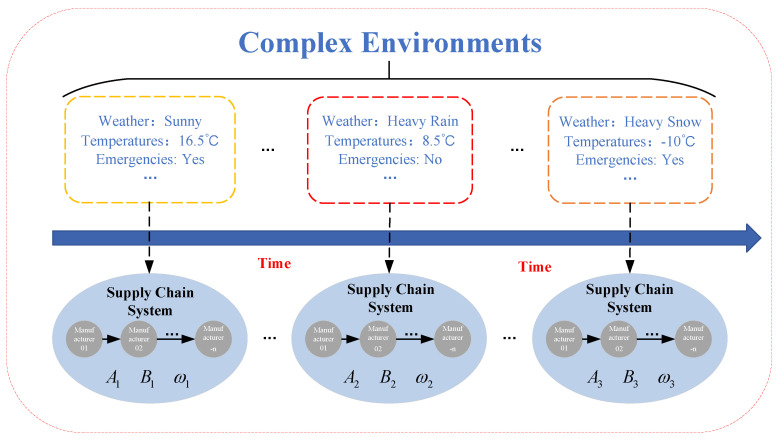
Operation diagram of perishable supply-chain system in complex environments.

Figure 6 shows the operation diagram of the supply-chain system under complex environments, where the complex environments are mainly reflected in the weather, average temperature, production conditions, and unexpected events during the operation cycle T. These parts will directly cause changes in the relevant parameters of the supply-chain system and market demand. The supply-chain system will exhibit different operating parameters in different environments, which often makes the management of the supply-chain system more difficult for enterprise managers.

According to Figure 6 and Equation (6), some parameters of the perishable supply-chain system are different under different environmental conditions, which can be described by the state-space equation as follows.

(9) $$\begin{array}{c} \left\{\begin{array}{c} x\left(k+1\right)=A_{i}x\left(k\right)+B_{i}u\left(k\right)+Dd\left(k\right)+D_{1}\omega_{i}\left(k\right)\\ y(k)=x(k)\\ z\left(k\right)=C_{1}x\left(k\right) \end{array}\right. \end{array}$$

where the parameters i=1,2,…,n represent different external environmental conditions.

Because the fixed market demand is known and constant, we set the controller into two parts:

(10) $$\begin{array}{c} u\left(k\right)=u_{1}\left(k\right)+u_{2}\left(k\right) \end{array}$$

the $u_{1}$ part is used to achieve robust $H_{\infty }$ state feedback control, and the $u_{2}$ part is used to balance the fixed market demand in the market. Then we can obtain

(11) $$\begin{array}{c} u_{2}\left(k\right)=-B_{i}^{-1}Dd\left(k\right). \end{array}$$

Combining Equations (9)–(11), we can obtain the dynamic equation of supply-chain inventory model with state feedback control in a complex environment.

(12) $$\begin{array}{c} \left\{\begin{array}{c} x\left(k+1\right)=A_{i}x\left(k\right)+B_{i}u_{1}\left(k\right)+D_{1}\omega_{i}\left(k\right)\\ y(k)=x(k)\\ z\left(k\right)=C_{1}x\left(k\right) \end{array}\right. \end{array}$$

For the constructed system (12), the system (12) is considered as an uncertain supply-chain system since each cycle *T* of the supply-chain system leads to uncertainty in parameters *A* and *B* under different operating environments. For the uncertain closed-loop supply-chain system (12), the following remarks are also required.

**Remark** **5.** *In the uncertain closed-loop supply-chain system* (12), $A_{i}$,$B_{i}$ *is the uncertainty parameter matrix of the system and satisfies*

(13) $$\begin{array}{ccc} A_{i}=\bar{A}+A^{*} & & B_{i}=\bar{B}+B^{*}, \end{array}$$

*where* $\bar{A}$ *and* $\bar{B}$ *are the initial values of the system parameters*, $A^{*}$ *and* $B^{*}$ *are the change matrices caused by the complex environmental changes*, *and we let*

(14) $$\left[\begin{array}{cc} A^{*} & B^{*} \end{array}\right]=HG\left[\begin{array}{cc} E_{1} & E_{2} \end{array}\right].$$

*Here, H, $E_{1}$, $E_{2}$ are matrices of known proper dimensions, and H, $E_{1}$, $E_{2}$ are related to the operating environment of the supply-chain system and its own operation. G is an unknown real matrix function and satisfies $G^{T}G⩽I$.*

**Remark** **6.** *From the uncertainty matrix of the system $A_{i}$,$B_{i}$ determined by the self-loss rate parameter ρ and the transport loss rate parameter δ, respectively. It can be seen that the parameters $A_{i}$,$B_{i}$ have a certain range of values.*

### 3.2. Robust $H_{\infty }$ Controller Design

In this part, we design the robust $H_{\infty }$ controller for perishable supply-chain system in complex environments based on the supply-chain system state-space Equation (12), which aims to help the supply-chain system to effectively adapt to the complex market environments, achieve the required operational demand, reduce the impact of demand fluctuations on the supply chain inventory costs, and improve the robustness of the supply-chain system in complex environments. The controller $u_{1}\left(k\right)$ is designed by $H_{\infty }$ state feedback so that the uncertain closed-loop supply-chain system (12) satisfies the following requirements.

- When the demand perturbation ω(k)=0, the uncertain closed-loop supply-chain inventory system (12) is capable of satisfying robust asymptotic stability.
- When the demand fluctuation ω(k)≠0, the uncertain closed-loop supply-chain system (12) should satisfy the $H_{\infty }$ performance parameter γ, which can be equated to the transfer function of ω(k)→z(k) in the system $G_{wz}$, then $G_{wz}$ is satisfied
  (15) $${∥G_{wz}∥}_{\infty }<\gamma ,$$
  where γ is the $H_{\infty }$ performance sub-optimization parameter of the system.

**Figure 7:**
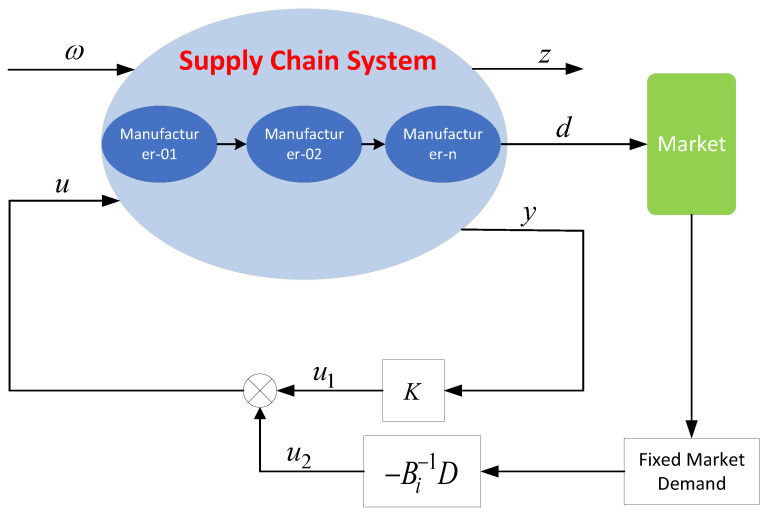
Robust $H_{\infty }$ controller design scheme based on state-space equation.

The design of the $H_{\infty }$ controller based on the state-space equation of the supply-chain system for uncertain supply-chain system in complex environments is shown in Figure 7, where the fixed market demand is obtained directly from the market survey, and the $H_{\infty }$ state feedback controller $u_{1}\left(k\right)$ is designed to make the uncertain closed-loop perishable supply chain system (12) satisfy requirement 1 and requirement 2.

From Figure 7, the control matrix of $H_{\infty }$ state feedback controller $u_{1}\left(k\right)$ is *K*, and then

(16) $$u_{1}\left(k\right)=Ky\left(k\right)=Kx\left(k\right)$$

By combining Equations (12), (13) and (16), we can obtain

(17) $$\begin{array}{c} \left\{\begin{array}{c} zx\left(z\right)=\left(\bar{A}+A^{*}\right)x\left(z\right)+\left(\bar{B}+B^{*}\right)Kx\left(z\right)+D_{1}\omega \left(z\right)\\ y(z)=x(z)\\ z\left(z\right)=C_{1}x\left(z\right) \end{array}\right. \end{array}$$

and then from the Equation (17), the z(z) can be written as

(18) $$\begin{array}{c} z\left(z\right)=C_{1}\cdot {\left[zI-\left(\bar{A}+A^{*}\right)-\left(\bar{B}+B^{*}\right)K\right]}^{-1}D_{1}\omega_{i}\left(z\right)\\ =C_{1}\cdot {\left(zI-A_{b}-HGE\right)}^{-1}D_{1}\omega \left(z\right) \end{array}$$

where $A_{b}=\bar{A}+\bar{B}K$, $E=E_{1}+E_{2}K$. Futhermore, with Equation (18), we can equate requirement 2 to

(19) $$\begin{array}{c} {∥G_{wz}∥}_{\infty }=\frac{{∥z(z)∥}_{\left[0,+\infty \right)}}{{∥\omega (z)∥}_{\left[0,+\infty \right)}}={∥C_{1}{\left(zI-A_{b}-HGE\right)}^{-1}D_{1}∥}_{\infty }<\gamma . \end{array}$$

Next, it is required that we introduce Lemma 1 in order to be able to develop controllers that fulfill the robust $H_{\infty }$ performance of perishable supply-chain systems in complicated environments.

**Lemma** **1** ([42])**.** *For the closed-loop system transfer function $G\left(z\right)=C{\left(zI-A\right)}^{-1}B$, where A is the stable matrix, then the necessary and sufficient condition for ${∥G(z)∥}_{\infty }<1$ is Riccati inequality*

(20) $$A^{T}XA-X+A^{T}XB{\left(I-B^{T}XB\right)}^{-1}B^{T}XA+C^{T}C<0$$

*and there exists a positive definite solution $X=X^{T}$, which makes $B^{T}XB<I$.*

Based on Lemma 1, we propose the Theorem 1.

**Theorem** **1.** *The transfer function of the closed-loop system is $G\left(z\right)=C{\left(zI-A-A_{1}\right)}^{-1}B$, where $A+A_{1}$ is the stable matrix. If there is a positive definite matrix $P=P^{T}$, which means the matrix inequality satisfies*

(21) $$\begin{array}{c} B^{T}PB<\gamma^{2}I\\ {\left(A+A_{1}\right)}^{T}P\left[B{\left(\gamma^{2}I-B^{T}PB\right)}^{-1}B^{T}+P^{-1}\right]P\left(A+A_{1}\right)-P+C^{T}C<0 \end{array}$$

*then the closed-loop system is robustly stable and meets ${∥G(z)∥}_{\infty }<\gamma$.*

The proof of Theorem 1 proceeds as follows.

**Proof.**  According to Lemma 1, a sufficient condition for ${∥G(z)∥}_{\infty }<1$ is Riccati’s inequality as shown in Equation (12). Then, for $H_{\infty }$ performance γ- suboptimization objective, that is, ${∥G(z)∥}_{\infty }<\gamma$, use $B_{\gamma }=\gamma^{-1}B$ instead of the matrix *B* in Lemma 1. In addition, taking $A=A+A_{1}$, then we can obtain

(22) $$\begin{array}{c} {\left(A+A_{1}\right)}^{T}P\left(A+A_{1}\right)-P+\\ {\left(A+A_{1}\right)}^{T}P\gamma^{-1}B{\left(I-\gamma^{-1}B^{T}P\gamma^{-1}B\right)}^{-1}\cdot \gamma^{-1}B^{T}P\left(A+A_{1}\right)+C^{T}C<0. \end{array}$$

Theorem 1 is true and the proof is complete by organizing Equation (22). □

According to Theorem 1, for the uncertain closed-loop supply-chain system (12), this paper proposes Theorem 2.

**Theorem** **2.** *The transfer function for the uncertain closed-loop supply-chain inventory system (12) is $G\left(z\right)=C_{1}{\left(zI-A_{b}-HGE\right)}^{-1}D_{1}$, where $A_{b}+HGE$ is a stable matrix for all permissible uncertain parameters. If there exists a scalar parameter α>0 and a positive definite matrix $P=P^{T}$, for all supply-chain inventory systems in complex environments, the following two conditions*

(23) $$\begin{array}{c} \alpha H^{T}LH<I\\ {\bar{A}}^{T}J\bar{A}+\alpha^{-1}E_{1}^{T}E_{1}+C_{1}^{T}C_{1}-N^{T}Q^{-1}N-P<0 \end{array}$$

*are satisfied simultaneously, where*

$$\begin{array}{c} M={\left(\gamma^{2}I-D_{1}^{T}PD_{1}\right)}^{-1}\\ L=P+PD_{1}MD_{1}^{T}P\\ J=L+LH{\left(\alpha^{-1}I-H^{T}LH\right)}^{-1}H^{T}L^{T}\\ N=\alpha^{-1}E_{2}^{T}E_{1}+{\bar{B}}^{T}J\bar{A}\\ Q=\alpha^{-1}E_{2}^{T}E_{2}+{\bar{B}}^{T}J\bar{B}. \end{array}$$

*Then the uncertain closed-loop supply-chain inventory system (12) is robust and stable and satisfies ${∥G(z)∥}_{\infty }<\gamma$. Then, the feedback control law is*

(24) $$\begin{array}{c} K=-Q^{-1}N=-(\alpha^{-1}E_{2}^{T}E_{2}+{\bar{B}}^{T}J\bar{B}{)}^{-1}\cdot \left(\alpha^{-1}E_{2}^{T}E_{1}+{\bar{B}}^{T}J\bar{A}\right). \end{array}$$

Next, we will prove Theorem 2.

**Proof.**  We set $L=P+PD_{1}MD_{1}^{T}P$, where $M={\left(\gamma^{2}I-D_{1}^{T}PD_{1}\right)}^{-1}$, and since *P* is known to be a positive definite symmetric matrix, we can obtain that both *L* and *M* are symmetric matrices. Then, the $A_{b}+HGE$ stability matrix is substituted into Lemma 1, the Equation (20) can be transformed into

(25) $$\begin{array}{c} {\left(A_{b}+HGE\right)}^{T}P\left[D_{1}\left(\gamma^{2}I-D_{1}^{T}PD_{1}\right)D_{1}^{T}+P^{-1}\right]P\left(A_{b}+HGE\right)\\ ={\left(A_{b}+HGE\right)}^{T}\left[PD_{1}MD_{1}^{T}P+P\right]\left(A_{b}+HGE\right)={\left(A_{b}+HGE\right)}^{T}L\left(A_{b}+HGE\right)\\ =A_{b}^{T}LA_{b}+E^{T}G^{T}H^{T}LHGE+E^{T}G^{T}H^{T}LA_{b}+A_{b}^{T}LHGE. \end{array}$$

Because of the state feedback control for the uncertain closed-loop supply chain system (12), it contains the corresponding uncertain parameters. Then we set the existing parameter matrices $W_{1}$, $W_{2}$, and scalar parameters α>0, and $W_{1}$, $W_{2}$ are defined as

(26) $$\begin{array}{c} W_{1}=\alpha^{\frac{1}{2}}{A_{b}}^{T}LHF^{-\frac{1}{2}}\;\;\;\;\;\;\;\;\;\;\;\;W_{2}=\alpha^{-\frac{1}{2}}E^{T}G^{T}F^{\frac{1}{2}}, \end{array}$$

where *F* is the symmetric matrix. By Equation (26) we obtain

(27) $$\begin{array}{c} W=\left(W_{1}-W_{2}\right)\cdot {\left(W_{1}-W_{2}\right)}^{T}=W_{1}W_{1}^{T}+W_{2}W_{2}^{T}-W_{1}W_{2}^{T}-W_{2}W_{1}^{T}\\ =\alpha A_{b}^{T}LHF^{-1}H^{T}L^{T}A_{b}+\alpha^{-1}E^{T}G^{T}FGE-E^{T}G^{T}H^{T}LA_{b}-A_{b}^{T}LHGE\geq 0. \end{array}$$

According to Equation (27), we have

(28) $$\begin{array}{c} \alpha A_{b}^{T}LHF^{-1}H^{T}L^{T}A_{b}+\alpha^{-1}E^{T}G^{T}FGE⩾E^{T}G^{T}H^{T}LA_{b}+A_{b}^{T}LHGE. \end{array}$$

Substituting Equation (28) into Equation (25), the Equation (25) can be translated as

(29) $$\begin{array}{c} {\left(A_{b}+HGE\right)}^{T}L\left(A_{b}+HGE\right)=A_{b}^{T}LA_{b}+E^{T}G^{T}H^{T}LHGE+E^{T}G^{T}H^{T}LA_{b}+A_{b}^{T}LHGE\\ \leq A_{b}^{T}LA_{b}+E^{T}G^{T}H^{T}LHGE+\alpha A_{b}^{T}LHF^{-1}H^{T}L^{T}A_{b}+\alpha^{-1}E^{T}G^{T}FGE\\ =A_{b}^{T}\left[L+\alpha LHF^{-1}H^{T}L^{T}\right]A_{b}+E^{T}G^{T}\left[H^{T}LH+\alpha^{-1}F\right]GE. \end{array}$$

We set *F* as

(30) $$F=(I-\alpha H^{T}LH),$$

then, by substituting Equation (30) into Equation (29), we can obtain

(31) $$\begin{array}{c} A_{b}^{T}\left[L+\alpha LHF^{-1}H^{T}L^{T}\right]A_{b}+E^{T}G^{T}\left[H^{T}LH+\alpha^{-1}F\right]GE\\ =A_{b}^{T}\left[L+\alpha LH{\left(I-\alpha H^{T}LH\right)}^{-1}H^{T}L^{T}\right]A_{b}+E^{T}G^{T}\alpha^{-1}IGE\\ =A_{b}^{T}JA_{b}^{T}+\alpha^{-1}E^{T}G^{T}GE, \end{array}$$

where $J=L+\alpha LH{\left(I-\alpha H^{T}LH\right)}^{-1}H^{T}L^{T}$, then we find *J* is a symmetric matrix. From the description of $G^{T}G⩽I$ in Remark 5, then from Equation (31) we obtain

(32) $$\begin{array}{c} A_{b}^{T}JA_{b}+\alpha^{-1}E^{T}G^{T}GE⩽A_{b}^{T}JA_{b}+\alpha^{-1}E^{T}E. \end{array}$$

Due to $A_{b}=\bar{A}+\bar{B}K$, $E=E_{1}+E_{2}K$, we have

(33) $$\begin{array}{c} A_{b}^{T}JA_{b}+\alpha^{-1}E^{T}E=A_{b}^{T}JA_{b}+\alpha^{-1}{\left(E_{1}+E_{2}K\right)}^{T}\left(E_{1}+E_{2}K\right)\\ ={\bar{A}}^{T}J\bar{A}+{\bar{A}}^{T}J\bar{B}K+k^{T}{\bar{B}}^{T}J\bar{A}+K^{T}{\bar{B}}^{T}J\bar{B}K\\ +\alpha^{-1}\left[E_{1}^{T}E_{1}+E_{1}^{T}E_{2}K+K^{T}E_{2}^{T}E_{1}+K^{T}E_{2}^{T}E_{2}K\right]\\ ={\bar{A}}^{T}J\bar{A}+K^{T}\left(\alpha^{-1}E_{2}^{T}E_{1}+{\bar{B}}^{T}J\bar{A}\right)+\alpha^{-1}E_{1}^{T}E_{1}\\ +\left({\bar{A}}^{T}J\bar{B}+\alpha^{-1}E_{1}^{T}E_{2}\right)K+K^{T}\left(\alpha^{-1}E_{2}^{T}E_{2}+{\bar{B}}^{T}J\bar{B}\right)K. \end{array}$$

Since we need to solve for the state feedback parameter matrix *K* that satisfies the system control conditions, we organize the Equation (33) by

(34) $$\begin{array}{c} N=\alpha^{-1}E_{2}^{T}E_{1}+{\bar{B}}^{T}J\bar{A}\;\;\;\;\;\;\;\;\;Q=\alpha^{-1}E_{2}^{T}E_{2}+{\bar{B}}^{T}J\bar{B}. \end{array}$$

In summary, according to the Equations (21), (33) and (34), the

(35) $$\begin{array}{c} {\left(A_{b}+HGE\right)}^{T}\left[PD_{1}{\left(\gamma^{2}I-{D_{1}}^{T}PD_{1}\right)}^{-1}{D_{1}}^{T}P+P\right]\left(A_{b}+HGE\right)-P+C_{1}^{T}C_{1}\\ ⩽A_{b}^{T}JA_{b}^{T}+\alpha^{-1}E^{T}E-P+C_{1}^{T}C_{1}\\ ={\bar{A}}^{T}J\bar{A}+\alpha^{-1}E_{1}^{T}E_{1}+K^{T}N+N^{T}K+K^{T}QK-P+C_{1}^{T}C_{1} \end{array}$$

can be obtained.

According to Theorem 1, we know that the uncertain closed-loop supply chain inventory system (12) is robust and stable as long as there exists a positive definite symmetric matrix *P* such that

(36) $$\begin{array}{c} {\left(A_{b}+HGE\right)}^{T}\left[PD_{1}{\left(\gamma^{2}I-{D_{1}}^{T}PD_{1}\right)}^{-1}{D_{1}}^{T}P+P\right]\left(A_{b}+HGE\right)-P+C_{1}^{T}C_{1}\\ ⩽A_{b}^{T}JA_{b}^{T}+\alpha^{-1}E^{T}E-P+C_{1}^{T}C_{1}\\ ={\bar{A}}^{T}J\bar{A}+\alpha^{-1}E_{1}^{T}E_{1}+K^{T}N+N^{T}K+K^{T}QK-P+C_{1}^{T}C_{1}<0 \end{array}$$

satisfies the condition. Then, we have

(37) $$\begin{array}{c} P>{\bar{A}}^{T}J\bar{A}+\alpha^{-1}E_{1}^{T}E_{1}+K^{T}N+N^{T}K\;+K^{T}QK+C_{1}^{T}C_{1}=f\left(K\right) \end{array}$$

By the condition 1 in Theorem 1, we can find that the size of the $H_{\infty }$ performance γ parameter is equivalent to the size of the positive semidefinite part of the symmetric positive definite matrix *P*. That is, if we minimize the $H_{\infty }$ performance γ parameter, it is equivalent to minimizing the positive semidefinite part of the symmetric positive definite matrix *P*. Therefore, if we want to make the symmetric positive definite matrix *P* as small as possible to improve the performance of $H_{\infty }$, we need to satisfy max{f(K)} as small as possible. Function f(K) can be expressed as

(38) $$\begin{array}{c} f\left(K\right)={\bar{A}}^{T}J\bar{A}+\alpha^{-1}E_{1}^{T}E_{1}+C_{1}^{T}C_{1}+K^{T}N+N^{T}K+K^{T}QK\\ ={\bar{A}}^{T}J\bar{A}+\alpha^{-1}E_{1}^{T}E_{1}+C_{1}^{T}C_{1}-N^{T}Q^{-1}N+{\left[K+Q^{-1}N\right]}^{T}Q\left[K+Q^{-1}N\right], \end{array}$$

then, we can find only when $K=-Q^{-1}N$, function f(K) obtains the maximum value. In this situation

(39) $$f_{\max}\left(K\right)={\bar{A}}^{T}J\bar{A}+\alpha^{-1}E_{1}^{T}E_{1}+C_{1}^{T}C_{1}-N^{T}Q^{-1}N.$$

Therefore Theorem 2 holds, and the proof is over. □

In conclusion, this paper uses Theorem 2 to create a state feedback controller for an uncertain closed-loop supply-chain system (12) in complex environments. The goal is to achieve the $H_{\infty }$ performance γ-suboptimization objective of a perishable supply-chain system in complex environments with unknown parameters within a specific range.

### 3.3. Real-Time Robust Optimization Principles

**Figure 8:**
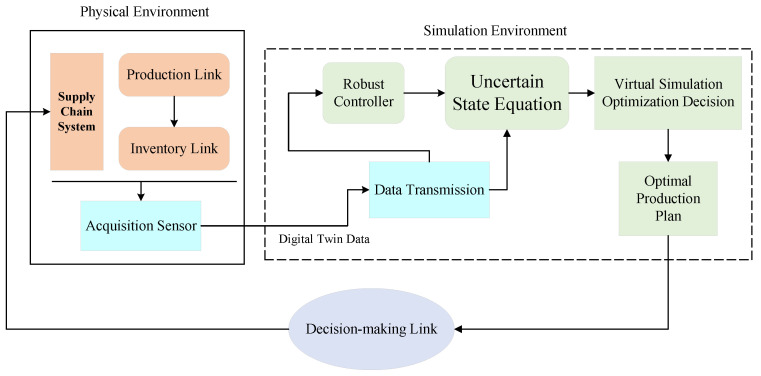
Real-time robust optimization principle based on supply-chain digital twins.

Perishable supply-chain systems in complex environments are influenced by many factors, making it impossible for enterprise managers to make reasonable and accurate production plans. The supply-chain digital twins can interact with the real supply-chain system in real-time, and obtain the operation status and environment status of the supply-chain system in real-time, and simulate it in the virtual simulation space, so as to help enterprise managers to make the optimal production plan and help enterprises adapt to the complex market environments. The working principle of the real-time robust optimization of supply-chain system based on digital twins is shown in Figure 8 below. The real-time robust optimization system based on the digital twins of the supply-chain system consists of a physical environment and a virtual environment, of which the physical environment and the virtual environment have been described above. From Figure 8, it can be seen that the robust optimization principle based on the digital twins of the supply-chain system is divided into two main parts. One of them is to use the acquisition sensors to collect the operational state data of the supply-chain system in the physical environment, to construct the dynamic equation of the uncertain supply-chain inventory system, update the unknown parameter data, and correct the inventory quantity of each link of the supply-chain system in real-time for better design of robust controllers. Secondly, using the collected real-time data to simulate in the virtual space, the optimal control inputs are analyzed in real-time to help the enterprise managers to formulate the most reasonable production plans and issue the corresponding instructions through the decision-making level to realize the production scheduling of the supply-chain system.

**Figure 9:**
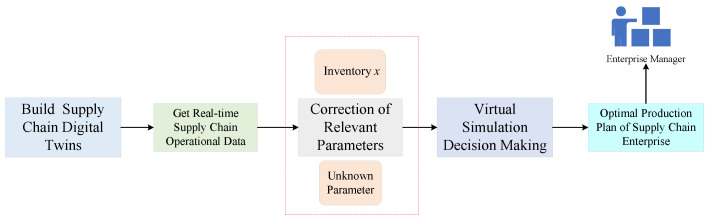
Real-time robust optimization flow chart of supply-chain digital twins.

By using the digital twin for real-time robust optimization of the supply-chain system in complex environments, the supply-chain system, the twin data, and the virtual simulation entity are connected as a whole, and the production plan of the supply-chain system in the complex environments can be optimized in real-time, as shown in Figure 9.

According to Figure 9, we can obtain the real-time robust optimization based on the supply-chain digital twins, and it can be divided into the following steps:

1. We construct the supply-chain digital twins by combining our supply chain state-space equation of the uncertain supply chain with digital twin technology;
2. The use of relevant sensors are used to collect twin data, including supply chain operation, status, and environmental conditions;
3. Based on the twin data collected in real-time, the parameters of the supply-chain system under the virtual simulation environment are updated and corrected, mainly including the real-time inventory quantity and unknown parameters *A* and *B* of the supply-chain enterprises;
4. The modified parameters are used for virtual simulation to develop the optimal production decision;
5. The optimal production decision is transmitted to the company manager through the data transmission layer.

From the above steps, it can be seen that the most critical step for real-time robust optimization of the supply-chain digital twins is step 3, which can make real-time corrections to the relevant parameters in the simulation environment to ensure that better production decisions can be obtained. According to the Equation (16), the controller input is related to the current enterprise inventory *x* and the state feedback control matrix *K*, so the supply-chain digital twins are used to make corrections from these two aspects. In this paper, we take advantage of the real-time data acquisition feature of the supply-chain digital twin to determine the parameters $E_{1}$ and $E_{2}$ in real-time. In this paper, the parameters $E_{1}$ and $E_{2}$ are corrected in real-time by taking advantage of the real-time data acquisition feature of the supply-chain digital twins, and let the corrected parameters be $\tilde{E_{1}}$ and $\tilde{E_{2}}$.

In summary, we can obtain the dynamic equation of the supply-chain system after the correction using the supply-chain digital twins as

(40) $$\left\{\begin{array}{c} \tilde{x}\left(k+1\right)=\tilde{A}\tilde{x}\left(k\right)+\tilde{B}{\tilde{u}}_{1}\left(k\right)+D_{1}\omega \left(k\right)\\ \tilde{y}\left(k\right)=\tilde{x}\left(k\right)\\ \tilde{z}\left(k\right)=C_{1}\tilde{x}\left(k\right)\\ {\tilde{u}}_{1}\left(k\right)=\tilde{K}\tilde{x}\left(k\right)\\ \tilde{K}=-{\left(\alpha^{-1}{\tilde{E}}_{2}^{T}{\tilde{E}}_{2}+{\bar{B}}^{T}J\bar{B}\right)}^{-1}\cdot \left(\alpha^{-1}{\tilde{E}}_{2}^{T}{\tilde{E}}_{1}+{\bar{B}}^{T}J\bar{A}\right). \end{array}\right.$$

## 4. Simulation and Comparative Analysis

**Figure 10:**
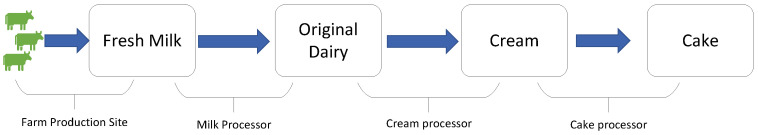
Production process of a cake product.

To verify the real-time robust optimization of perishable supply-chain system by constructing the digital twins of the supply-chain, this paper conducts a comparative simulation study based on the production and sale of a cake in the market, which contains multiple upstream producers, and its processing flow is shown in Figure 10. From the initial production of fresh milk at the cow production base to the downstream milk processor to produce raw dairy products, the raw dairy products are sent to the downstream cream processing manufacturer to produce cream, and finally the cream is made into cake products by the cake processor for sale. The structure is in line with the single-chain chain supply-chain system marketing model, and the processed products are also a type of perishable product, which is fully consistent with the needs of this paper to study the object of perishable supply-chain system.

Based on a cake processing process, to verify the applicability and superiority of the proposed method, two kinds of simulation experiments are conducted for the cake supply-chain system: traditional robust optimization based on the state-space equation and real-time robust optimization based on the digital twins of the supply-chain. Firstly, we obtain the relevant parameters of the cake supply chain system and construct the state-space equation of the supply-chain system. Secondly, we determine the variation range of the unknown parameters and give the performance parameter γ according to the previous experience, and use the Theorem 2 proposed in this paper to find out the state feedback matrix $H_{\infty }$ control of the supply-chain system under different simulation situations. Finally, using the inventory quantity as the control index, we analyze the trend of inventory quantity and inventory costs of the supply-chain system under complex environments.

From Figure 10, we can see that the upstream processors of the cake are 3, that is, we take n=3 in this paper. By analyzing the production–sales–storage data and making reasonable data assumptions about the cake supply-chain system, we can obtain the initial parameters of the three-stage supply-chain system model of the cake as follows:

$$\bar{A}=\left[\begin{array}{ccc} \;0.79 & \;0 & \;0\\ \;0 & \;0.86 & 0\\ \;0 & \;0 & \;0.91 \end{array}\right]\;\;\bar{B}=\left[\begin{array}{ccc} \;0.95 & \;-1 & \;0\\ \;0 & \;0.91 & \;-1\\ \;0 & \;0 & \;0.89 \end{array}\right]$$

$$D=\left[\begin{array}{c} 0\\ 0\\ -1 \end{array}\right]\;\;\;D_{1}=\left[\begin{array}{c} -1.35\\ -1.13\\ -1 \end{array}\right]$$

$$C_{1}=\left[\begin{array}{ccc} 1.51 & ;1.32 & ;1.18 \end{array}\right]\;\;\;d=\left[1.06\right].$$

It is known that the robust sub-optimization parameter γ=1.20 of the cake supply-chain system is taken in this paper, and the range of variation of the relevant parameters of the three-level supply-chain system can be obtained through previous investigations and expert experience as follows:

$$\omega \left(k\right)=\frac{1}{1+k^{2}}$$

$$A^{*}\left(k\right)=\left[\begin{array}{ccc} 0.08\sin(k) & 0 & 0\\ 0 & 0.06\sin(k) & 0\\ 0 & 0 & 0.02\sin(k) \end{array}\right]$$

$$B^{*}\left(k\right)=\left[\begin{array}{ccc} 0 & \;0 & 0\\ 0 & \;0.05\sin(k) & 0\\ 0 & \;0 & 0.07\sin(k) \end{array}\right].$$

According to Theorem 2, the matrix inequality (41) can be constructed, and the proof procedure is shown in Appendix A. By solving inequality (41), we can obtain the state feedback control law *K* for uncertain perishable supply-chain systems in complex environments.

(41) $$\begin{array}{c} \left[\begin{array}{cc} \begin{array}{c} {\bar{A}}^{T}J\bar{A}-P \end{array} & \begin{array}{c} \bar{A}J{\bar{B}}^{T} \end{array}\\ {\bar{B}}^{T}J\bar{A} & {\bar{B}}^{T}J\bar{B} \end{array}\right]{\left[\begin{array}{cc} \begin{array}{c} \alpha^{-1}E_{1}^{T}E_{1}+C_{1}^{T}C_{1} \end{array} & \begin{array}{c} \alpha^{-1}E_{1}E_{2}^{T} \end{array}\\ \alpha^{-1}E_{2}^{T}E_{1} & \alpha^{-1}E_{2}^{T}E_{2} \end{array}\right]}^{-1}+\left[\begin{array}{cc} I & *\\ {}* & I \end{array}\right]<0\\ J={\left(P^{-1}-\gamma^{-2}D_{1}D_{1}^{T}-\alpha HH^{T}\right)}^{-1} \end{array}$$

In this paper, we take γ=1.20, α=0.1, and use the LMI toolbox to solve matrix inequality (41), and we can obtain the state feedback matrix *K* as

$$K=\left[\begin{array}{ccc} -0.6295 & 5.2289 & 2.8302\\ -0.2908 & 2.4152 & -1.3072\\ 0.3018 & -2.5072 & 1.3570 \end{array}\right]\times 10^{-2}.$$

A robust $H_{\infty }$ controller for a perishable supply-chain system in a complex environments is designed using the solved state feedback matrix *K*, and a conventional robust optimization simulation experiment based on the state-space equation of the uncertain supply-chain is conducted with the following initial parameters

$$x\left(0\right)=\left[\begin{array}{c} 8\\ 5\\ 6 \end{array}\right]\;\;\;u\left(0\right)=\left[\begin{array}{c} 0\\ 0\\ 0 \end{array}\right].$$

**Figure 11:**
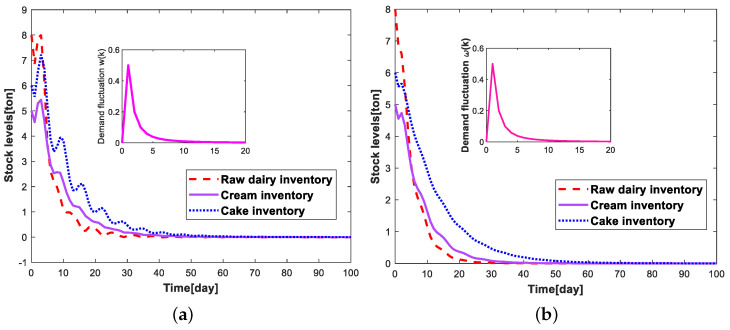
Comparison chart of inventory under different experimental conditions. (**a**) Without supply-chain digital twins; (**b**) With supply-chain digital twins.

**Figure 12:**
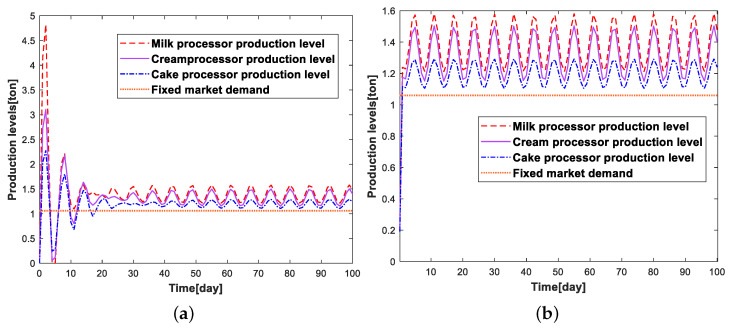
Comparison chart of production levels under different experimental conditions. (**a**) Without supply-chain digital twins; (**b**) With supply-chain digital twins.

**Figure 13:**
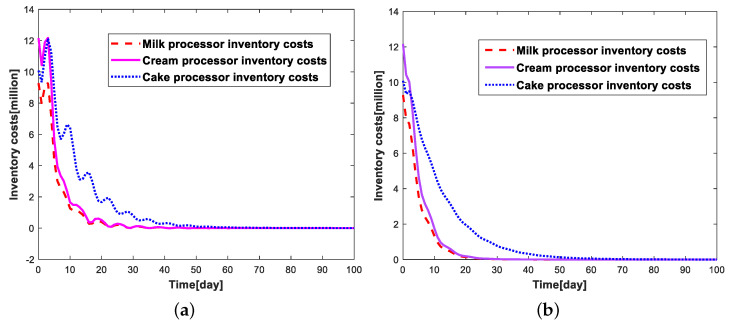
Comparison chart of inventory costs under different experimental conditions. (**a**) Without supply-chain digital twins; (**b**) With supply-chain digital twins.

According to the above experimental conditions, the comparison of the change of inventory levels, production levels, and inventory costs of each link of the cake supply chain system under the two simulation conditions is shown in Figure 11, Figure 12, and Figure 13, respectively.

By analyzing Figure 11, we can find that the simulation process can update the relevant parameters in real-time due to the existence of the supply chain digital twins, which can develop a more reasonable production strategy compared with the traditional robust optimization, and greatly reduce the inventory fluctuation in each link of the cake supply-chain system. When the supply-chain system is stable, the inventory quantity of each link of the supply-chain system will gradually converge to 0, maintaining the dynamic stability of production and sales, ensuring market demand, and reducing the inventory costs of the enterprise caused by inventory. Through Figure 12, we can also find that the real-time robust optimization based on the digital twins of the supply-chain can help companies develop smoother production strategies, while the production strategies developed by the traditional robust optimization are more volatile. Since the inventory cost of each link of the cake supply-chain system is proportional to the inventory quantity, we can find that the trend of the change curve of inventory costs are the same as the trend of the change of inventory quantity in Figure 13.

**Figure 14:**
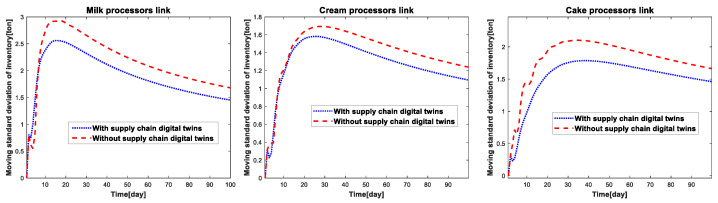
Moving standard deviation of inventory in different parts of the cake supply-chain system.

**Figure 15:**
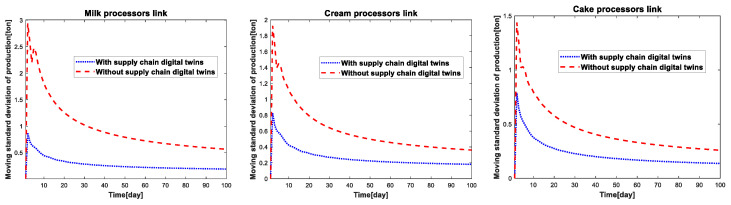
Moving standard deviation of production in different parts of the cake supply-chain system.

In order to better reflect the fluctuation trend of inventory quantity and production quantity of different links of the supply-chain system under different simulation conditions, this paper introduces the moving standard deviation index, where the moving standard deviation is defined as the standard deviation of each sampling point in the operation of the supply chain system to obtain the moving standard deviation. According to the definition, the moving standard deviation of inventory and the moving standard deviation of production for each link of the cake supply-chain system under two simulation conditions can be calculated as shown in Figure 14 and Figure 15.

From the analysis of Figure 14, we can see that compared with the traditional robust optimization, the real-time robust optimization based on the supply chain digital twins can reduce inventory fluctuation, reduce the production storage pressure and avoid the waste of resources in the enterprise. Similarly, Figure 15 reflects that with the help of real-time updating of the parameters of the supply-chain digital twins, the virtual simulation environment can formulate more reasonable production strategies based on the operation status of the supply-chain system, reduce the number of production fluctuations, and reduce the waste of resources and cost increase caused by the production.

In summary, in order to reflect the effectiveness and superiority of real-time robust optimization based on the supply-chain digital twins, a comprehensive comparison of the simulation results in two different cases is presented in Table 1.

## 5. Conclusions

In this paper, a solution based on supply-chain digital twins is proposed for the real-time robust optimization of a perishable supply-chain system in complex environments. The state-space of the supply-chain system is constructed based on the logical relationship of its operation, and the digital twin technology is used to build the supply-chain digital twins. The experimental results show that, compared with the traditional robust optimization, the real-time robust optimization solution based on supply-chain digital twins can update the relevant parameters in real-time based on the twin data generated from the operation state of the supply-chain system, which can help enterprises to formulate more appropriate production–storage–sales strategies to meet the market demand while reducing the enterprise’s inventory, reducing the waste of resources, and effectively help perishable supply chain system enterprises to adapt to the complex, volatile, and fluctuating market environments.

This paper provides a theoretical basis and feasible means for the managers of perishable supply-chain system enterprises to cope with the complex market environments. However, there are still some research deficiencies in this paper. The research on the supply-chain system does not consider the time-lag problem when the products are shipped, and further research can be conducted by introducing time-lag parameters. In addition, this paper considers that the supply-chain inventory system model is mainly linear, but there are many nonlinear factors in the real environment, so we can add relevant nonlinear relationships for further research in the future.

## Figures and Tables

**Table 1:** Comparison of different performance parameters in two simulation cases.

Simulation Situation	Cake Supply-Chain Link	Whether Shortage	Adjusting Duration [day]	MMSDI [ton]	MMSDP [ton]
Traditional robust optimization ( without digital twins)	Milk processor	Yes	50	2.9314	2.9402
	Cream processor	Yes	60	1.6925	1.9254
	Cake processor	No	60	2.0991	1.4389
Real-time robust optimization ( with digital twins)	Milk processor	No	30	2.5605	0.8759
	Cream processor	No	40	1.5825	0.8309
	Cake processor	No	60	1.7869	0.7886

MMSDI: maximum moving standard deviation of inventory; MMSDP: maximum moving standard deviation of production.

## Data Availability

This article does not use public data. All data is provided by enterprises and cannot be shared.

## Appendix A

In order to be able to prove Equation (41) in the main text, the following lemma needs to be introduced.

**Lemma** **A1** ([43])**.** *For an uncertain closed-loop system as shown in Equation (A1), if there exists a state feedback u(k)=Kx(k) and a reversible symmetric positive definite matrix $P\in R^{n\times n}$, the following inequalities hold simultaneously:*

(A1) $$\left\{\begin{array}{c} x\left(k+1\right)=\left(A_{c}+HGE_{c}\right)x\left(k\right)+B_{1}\omega \left(k\right)\\ y(k)=Cx(k) \end{array}\right.$$

(A2) $$\begin{array}{c} B_{1}^{T}PB_{1}<\gamma^{2}I\\ {\left(A_{c}+HGE_{c}\right)}^{T}{\left(P^{-1}-\gamma^{-2}B_{1}B_{1}^{T}\right)}^{-1}\left(A_{c}+HGE_{c}\right)-P+C^{T}C<0 \end{array}$$

**Lemma** **A2** ([44])**.** *(Schur complementary lemma) (Ref.) For a symmetric matrix $S=\left[\begin{array}{cc} \begin{array}{c} S_{11}\\ \; \end{array} & \begin{array}{c} S_{12}\\ \; \end{array}\\ \;S_{12}^{T} & S_{22} \end{array}\right]$, the following conditions are equivalent.*

$$\begin{array}{c} (1)S<0\\ \left(2\right)S_{11}<0,S_{22}-S_{12}^{T}S_{11}^{-1}S_{12}<0\\ \left(3\right)S_{22}<0,S_{11}-S_{12}S_{22}^{-1}S_{12}^{T}<0 \end{array}$$

According to the Lemmas A1 and A2, the proof of Equation (41) is as follows:

From Lemma A1 it follows that

(A3) $$\begin{array}{c} {\left(P^{-1}-\gamma^{-2}B_{1}B_{1}^{T}\right)}^{-1}=P+PB_{1}{\left(\gamma^{2}I-B_{1}^{T}PB_{1}\right)}^{-1}B_{1}^{T}P. \end{array}$$

Then we can get

(A4) $$\begin{array}{c} L=P+PD_{1}{\left(\gamma^{2}I-D_{1}^{T}PD_{1}\right)}^{-1}D_{1}^{T}P={\left(P^{-1}-\gamma^{-2}D_{1}D_{1}^{T}\right)}^{-1}, \end{array}$$

then

(A5) $$\begin{array}{c} J=L+LH{\left(\alpha^{-1}I-H^{T}LH\right)}^{-1}H^{T}L^{T}={\left(L^{-1}-\alpha HH^{T}\right)}^{-1}\\ ={\left(P^{-1}-\gamma^{-2}D_{1}D_{1}^{T}-\alpha HH^{T}\right)}^{-1}. \end{array}$$

From Theorem 2 and Lemma A1 we can get the form of transforming the two conditions in Lemma 2 into matrix inequalities as shown below:

(A6) $$\begin{array}{c} \left[\begin{array}{cc} \begin{array}{c} {\bar{A}}^{T}J\bar{A}+\alpha^{-1}E_{1}^{T}E_{1}+C_{1}^{T}C_{1}-P \end{array} & \begin{array}{c} {\left(\alpha^{-1}E_{2}^{T}E_{1}+{\bar{B}}^{T}J\bar{A}\right)}^{T} \end{array}\\ \alpha^{-1}E_{2}^{T}E_{1}+{\bar{B}}^{T}J\bar{A} & \alpha^{-1}E_{2}^{T}E_{2}+{\bar{B}}^{T}J\bar{B} \end{array}\right]\\ =\left[\begin{array}{cc} \begin{array}{c} {\bar{A}}^{T}J\bar{A}-P \end{array} & \begin{array}{c} \bar{A}J{\bar{B}}^{T} \end{array}\\ {\bar{B}}^{T}J\bar{A} & {\bar{B}}^{T}J\bar{B} \end{array}\right]+\left[\begin{array}{cc} \begin{array}{c} \alpha^{-1}E_{1}^{T}E_{1}+C_{1}^{T}C_{1} \end{array} & \begin{array}{c} \alpha^{-1}E_{1}E_{2}^{T} \end{array}\\ \alpha^{-1}E_{2}^{T}E_{1} & \alpha^{-1}E_{2}^{T}E_{2} \end{array}\right]<0. \end{array}$$

Then Equation (A6) can be transformed into

(A7) $$\begin{array}{c} \left[\begin{array}{cc} \begin{array}{c} {\bar{A}}^{T}J\bar{A}-P \end{array} & \begin{array}{c} \bar{A}J{\bar{B}}^{T} \end{array}\\ {\bar{B}}^{T}J\bar{A} & {\bar{B}}^{T}J\bar{B} \end{array}\right]{\left[\begin{array}{cc} \begin{array}{c} \alpha^{-1}E_{1}^{T}E_{1}+C_{1}^{T}C_{1} \end{array} & \begin{array}{c} \alpha^{-1}E_{1}E_{2}^{T} \end{array}\\ \alpha^{-1}E_{2}^{T}E_{1} & \alpha^{-1}E_{2}^{T}E_{2} \end{array}\right]}^{-1}+\left[\begin{array}{cc} I & *\\ {}* & I \end{array}\right]<0 \end{array}$$

That is, we can obtain the proof of Equation (41).
